# Comparative Analysis of Short-Term and Long-Term Clinical Efficacy of Mesenchymal Stem Cells from Different Sources in Knee Osteoarthritis: A Network Meta-Analysis

**DOI:** 10.1155/2024/2741681

**Published:** 2024-05-31

**Authors:** Qi Xin Ding, Xu Wang, Tian Shu Li, Yue Fang Li, Wan Yue Li, Jia Huan Gao, Yu Rong Liu, WeiSheng Zhuang

**Affiliations:** ^1^ Henan University of Chinese Medicine, Zhengzhou, China; ^2^ Henan University, Zhengzhou, China; ^3^ First Affiliated Hospital of Jinan University, Guangzhou, China; ^4^ Henan Provincial People's Hospital, Zhengzhou, China; ^5^ Shandong First Medical University, Jinan, China

## Abstract

**Background:**

Joint articular injection of mesenchymal stem cells (MSCs) has emerged as a novel treatment approach for osteoarthritis (OA). However, the effectiveness of MSCs derived from different sources in treating OA patients remains unclear. Therefore, this study aimed to explore the differences between the effectiveness and safety of different sources of MSCs.

**Materials and Methods:**

For inclusion consideration, we searched trial registries and published databases, including PubMed, Cochrane Library, Embase, and Web of Science databases. Revman (V5.3), STATA (V16.0), and R (V4.0) were utilized for conducting data analysis, while the Cochrane Risk of Bias Tool was employed for assessing the quality of the studies. We derived outcome measures at 6 and 12 months based on the duration of study follow-up, including visual analog scale (VAS) score, WOMAC score, WOMAC pain, WOMAC Functional Limitation, and WOMAC stiffness. The evaluation time for short-term effectiveness is set at 6 months, while 12 months is utilized as the longest follow-up time for most studies to assess long-term effectiveness.

**Results:**

The evaluation of literature quality showed that the included studies had excellent methodological quality. A meta-analysis revealed that different sources of MSCs improved knee function and pain more effectively among patients suffering from knee OA (KOA) than controls. The results of the network meta-analysis showed the following: short-term functional improvement (the indexes were evaluated after 6 months of follow-up) (WOMAC total score: bone marrow-derived MSC (BMMSC) vs. adipose-derived MSC (ADMSC) (mean difference (MD) = −20.12, 95% confidence interval (CI) −125.24 to 42.88), umbilical cord-derived MSC (UCMSC) (MD = −7.81, 95% CI −158.13 to 74.99); WOMAC stiffness: BMMSC vs. ADMSC (MD = −0.51, 95% CI −7.27 to 4.29), UCMSC (MD = −0.75, 95% CI −9.74 to 6.63); WOMAC functional limitation: BMMSC vs. ADMSC (MD = −12.22, 95% CI −35.05 to 18.86), UCMSC (MD = −9.31, 95% CI −44.26 to 35.27)). Long-term functional improvement (the indexes were evaluated after 12 months of follow-up) (WOMAC total: BMMSC vs. ADMSC (MD = −176.77, 95% CI −757.1 to 378.25), UCMSC (MD = −181.55, 95% CI −937.83 to 541.13); WOMAC stiffness: BMMSC vs. ADMSC (MD = −0.5, 95% CI −26.05 to 18.61), UCMSC (MD = −1.03, 95% CI −30.44 to 21.69); WOMAC functional limitation: BMMSC vs. ADMSC (MD = −5.18, 95% CI −316.72 to 177.1), UCMSC (MD = −8.33, 95% CI −358.78 to 218.76)). Short-term pain relief (the indexes were evaluated after 6 months of follow-up) (VAS score: UCMSC vs. BMMSC (MD = −10.92, 95% CI −31.79 to 12.03), ADMSC (MD = −14.02, 95% CI −36.01 to 9.81), PLMSC (MD = −17.09, 95% CI −46.31 to 13.17); WOMAC pain relief: BMMSC vs. ADMSC (MD = −11.42, 95% CI −39.52 to 11.77), UCMSC (MD = −6.73, 95% CI −47.36 to 29.15)). Long-term pain relief (the indexes were evaluated after 12 months of follow-up) (VAS score: BMMSC vs. UCMSC (MD = −4.33, 95% CI −36.81 to 27.08), ADMSC (MD = −11.43, 95% CI −37.5 to 13.42); WOMAC pain relief: UCMSC vs. ADMSC (MD = 0.23, 95% CI −37.87 to 38.11), BMMSC (MD = 5.89, 95% CI −25.39 to 51.41)). According to the GRADE scoring system, WOMAC, VAS, and AE scores were of low quality.

**Conclusion:**

Meta-analysis suggests MSCs can effectively treat KOA by improving pain and knee function compared to control groups. In terms of functional improvement in KOA patients, both short-term (6-month follow-up) and long-term (12-month follow-up) results indicated that while the differences between most treatments were not statistically significant, bone marrow-derived MSCs may have some advantages over other sources of MSCs. Additionally, BM-MSCs and UC-MSCs may offer certain benefits over ADMSCs in terms of pain relief for KOA patients, although the variances between most studies were not statistically significant. Therefore, this study suggests that BM-MSCs may present clinical advantages over other sources of MSCs.

## 1. Introduction

Osteoarthritis (OA) is a common chronic degenerative joint disease that mainly affects articular cartilage and surrounding tissues, leading to joint space narrowing and osteophyte formation. Weight-bearing joints, such as ankles, knees, and hips, are especially affected. During the initial phases, it presents itself as localized pain and rigidity, progressively resulting in a decline in physical abilities and impairment of function [1, 2]. In the past, OA was considered a simple wear-and-tear condition; however, it is now understood to be a complex multifactorial process involving inflammation and metabolic factors [3, 4, 5, 6, 7]. Knee OA (KOA) is a common type of OA. According to the Global Burden of Disease Study 2015, approximately 85% of the burden of OA is attributed to KOA, with an estimated prevalence of 10% in men aged > 60 years and 13% in women aged > 60 [8, 9]. Current clinical treatments for KOA include nonsteroidal anti-inflammatory drugs (NSAIDs), total knee replacement surgery, and intra-articular injections [10, 11, 12, 13]. However, NSAIDs provide pain relief but do not alleviate joint damage. While total knee replacement surgery has proven effective in treating KOA, it is expensive and burdensome, with potential complications such as infection, thrombosis, and the need for revision surgery, which increases the risk of mortality in elderly patients [14]. Recently, stem cell transplantation has emerged as a promising treatment option for OA, with significant advantages. Mesenchymal stem cells (MSCs), a type of multipotent adult stem cells, possess anti-inflammatory, immune-modulatory, and regenerative properties and have been widely used in cell therapy for various diseases. MSCs can be derived from bone marrow, adipose tissue, and umbilical cord and are referred to as bone marrow-derived MSCs (BMMSCs), adipose-derived MSCs (ADMSCs), and umbilical cord-derived MSCs (UCMSCs), respectively [15, 16, 17]. The main markers expressed by MSCs in the human body are CD90, CD73, and CD105, although slight differences exist in marker expression between stem cells from different tissues [12, 18]. MSCs have been used in the clinical treatment of OA with good results. Multiple studies have demonstrated the significant advantages of stem cells for pain relief, joint function improvement, and cartilage regeneration in patients with OA [19, 20, 21]. Most of the studies included patients with Kellgren–Lawrence classifications ranging from I to III, while some studies included patients with IV classifications and showed good efficacy in improving patients' symptoms [21, 22, 23]. However, owing to the differences in their origin and therapeutic effects, MSCs from different sources exhibit variations in clinical efficacy for treating KOA. The clinical efficacy of different sources of MSCs for the treatment of OA has been evaluated differently in previous studies, and the dosage of bone marrow, adipose, and UCMSCs commonly used in clinical practice and the selection of different cells are the current problems. Therefore, we reviewed the latest research reports on the treatment of KOA with MSCs from different sources and compared them with the control group, and indirectly compared MSCs from different sources through network meta-analysis, using the control group as an intermediate bridge to compare the treatment groups with different MSCs. So, the aim of this study was to evaluate the efficacy of MSCs from different sources and the differences between them, as well as to provide a more reliable and tailored reference for the therapeutic injection therapy of KOA.

## 2. Materials and Methods

### 2.1. Registration

The present study was a systematic review of published studies, which does not require the consent of patients or ethical approval [24]. According to the guidelines for preferred reporting items and previously published protocols, this review was based on systematic reviews and meta-analyses [25]. This systematic evaluation has been registered in PROSPERO (reference number: CRD 42023448217) as a detailed protocol.

### 2.2. Data Sources and Retrieval Strategy

We conducted a comprehensive search of trial registries and published databases, including PubMed, Embase, the Cochrane Library, and Web of Science. The search encompassed all randomized controlled trials (RCTs) on MSCs and OA published in these databases until February 15, 2024. A combination of subject terms and free text were used to construct the search model, which was then applied to the four aforementioned databases. Then a search was conducted in the database based on the following search terms: (“Mesenchymal Stem Cells” or “Stem Cell, Mesenchymal” or “Mesenchymal Stem Cell” or “Bone Marrow Mesenchymal Stem Cells” or “Adipose-Derived Mesenchymal Stem Cells” or “Wharton Jelly Cells” and “Osteoarthritis” or “Osteoarthritides” or “Osteoarthrosis” or “Osteoarthroses” or “Knee Osteoarthritides” or “Osteoarthritis of Knee.” The complete search formula is in *Supplementary 1*.

### 2.3. Inclusion and Exclusion Criteria

The inclusion and exclusion process for articles followed the principles of PICOS (Participants, Interventions, Comparisons, Outcomes, and Studies). Inclusion criteria were as follows: (i) studies with an RCT design, (ii) patients with KOA, (iii) experimental groups receiving at least one intra-articular injection of MSCs from any source, and (iv) include at least one of the following efficacy parameters, such as the University of Western Ontario and McMaster University Osteoarthritis (WOMAC) score or its subscale or visual analog scale (VAS) score. The exclusion criteria were as follows: (i) studies involving vascular stromal cells, (ii) studies with only abstracts available (insufficient data), and (iii) studies for which full-text access was not available.

### 2.4. Data Extraction and Literature Quality Evaluation

Two researchers (DQX and WX) extracted information from the included studies, including the publication authors, publication year, sample size, average age, intervention measures, outcome parameters, and follow-up time. For the efficacy indicators, outcome indices at 6 and 12 months of follow-up, including WOMAC total, WOMAC pain, WOMAC stiffness, WOMAC functional limitation, and VAS score. Data not included in the original document were extracted using specialized extraction tools. In each included study, two independent evaluators evaluated the methodological quality according to the Cochrane Collaboration guidelines. As a result of this tool, selection bias, allocation bias, implementation bias, reporting bias, follow-up bias, and other biases were assigned a “low,” “unclear,” or “high” risk.

### 2.5. Outcome Indicators

The majority of the studies under review primarily assessed outcomes at 6 and 12 months. Consequently, the 12-month outcomes were employed to assess long-term efficacy, while the 6-month outcomes were utilized to gauge short-term efficacy. Functional improvement was evaluated using WOMAC total, WOMAC stiffness, and WOMAC functional limitation scores, whereas pain relief was assessed using the VAS and WOMAC pain scores.

### 2.6. Statistical Analysis

In this study, Revman (V5.3), STATA (V16.0), and R (V4.0) were used for data analysis. First, traditional meta-analysis and network diagram plotting were performed using STATA (Stata is a statistical analysis tool with menu-driven operations that cannot be performed with conventional meta-analysis software). Second, the included studies were analyzed using a random-effects model and network meta-analysis using the GeMTC package in R (R is a statistical analysis software based on the Bayesian model). The level of study heterogeneity is quantified by *i*^2^. If *i*^2^ exceeds 50%, a random effects model will be employed. When *i*^2^ surpasses 75%, indicating substantial heterogeneity, sensitivity analysis will be conducted to identify the source of heterogeneity and assess the stability of the results, as well as compare the impact of various clinical factors. In cases where no underlying cause for heterogeneity is identified, descriptive analyses will be carried out. None of the studies formed a closed loop. Therefore, a consistency model is used, and no inconsistency testing is required. Finally, we utilize GRADE profiler software (https://gradeprofifiler.software.informer.com/) according to the index and analysis results of this study to evaluate the quality of the evidence, and using Stata software (version 16.0, http://www.stata.com) Construct a funnel plot to determine publication bias.

## 3. Results

### 3.1. Characteristics of Research

**Figure 1:**
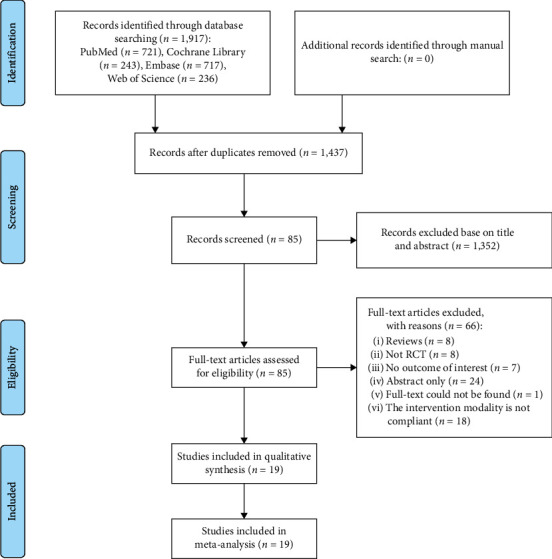
The flowchart of the literature search and screening process.

A total of 1,917 papers were found in four databases: 721 in PubMed, 243 in Cochrane Library, 717 in Embase, and 236 in Web of Science. After removing duplicates, 1,437 articles remained. Among these, 85 articles were selected for full-text reading based on their titles and abstracts. Of these, 66 were excluded (the intervention modality is not compliant: included vascular interstitial fraction, bone marrow aspirate, and adipose tissue-derived fraction), leaving 19 studies for the final analysis (Figure 1). These included eight studies on BMMSCs, seven on ADMSCs, three on UCMSCs, and one on placenta-derived MSCs (PLMSCs) (Table 1).

### 3.2. Literature Quality Evaluation Results

**Figure 2:**
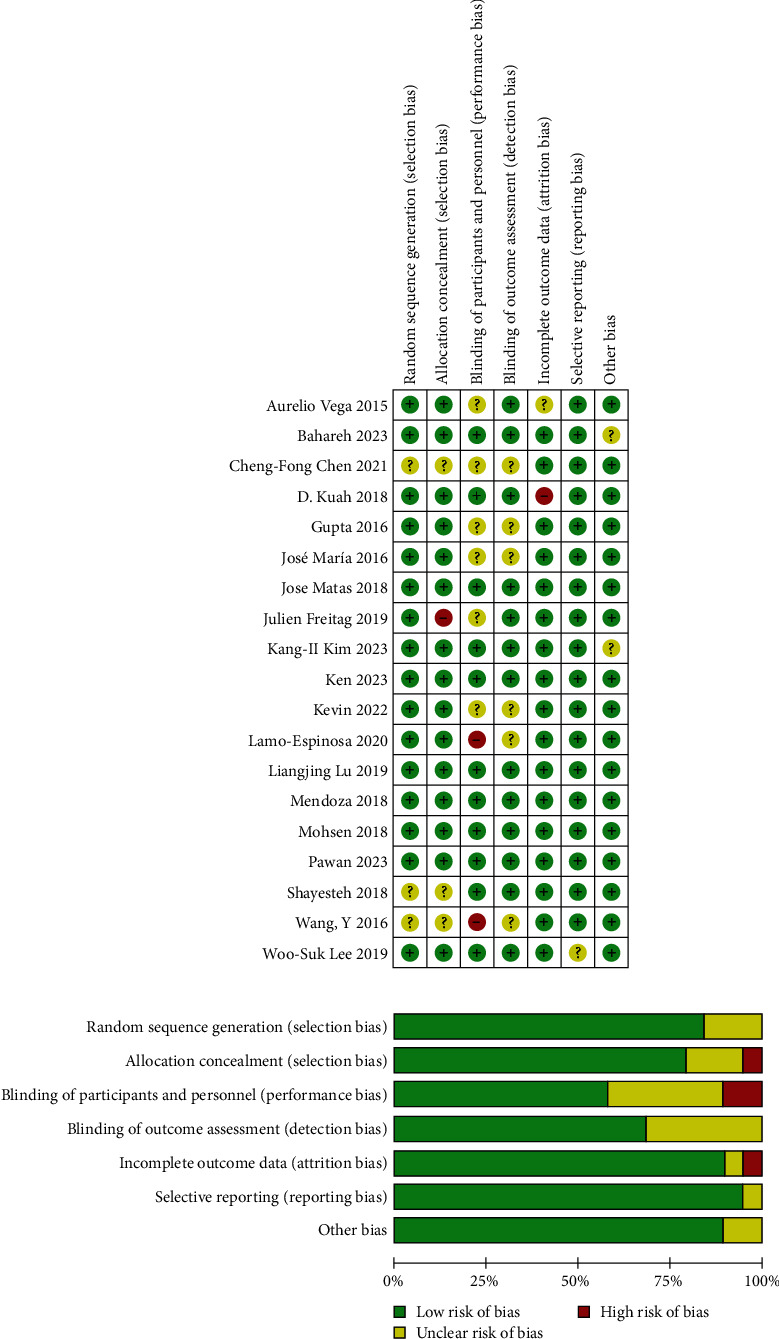
Risk of bias summary of RCTs. RCT, randomized controlled experiment.

A tool for evaluating the quality of RCTs, the Cochrane Risk of Bias Tool, was used to assess the quality of the 19 studies included in this analysis. Among these, four studies were assessed as having a low risk of bias. Regarding randomization, 16 studies described the random allocation methods employed in the research, whereas three studies did not provide detailed explanations of their specific randomization methods. Regarding data integrity, 17 studies explained the follow-up duration and excluded cases. The literature quality evaluation revealed that the included studies had excellent methodological quality in general. The risk of bias in each study is illustrated in Figure 2.

### 3.3. Convergence and Consistency Tests

In this study, a Bayesian model was constructed, and the results revealed minimal fluctuations in the Markov chain and a stable trajectory of iterations approaching a steady state. The probability of superiority was close to 1, indicating good model convergence. Therefore, a consistency model was adopted for network meta-analysis. No closed loop was formed between the various intervention measures; thus, no inconsistency test was required.

### 3.4. Outcomes Evaluation

#### 3.4.1. Functional Improvement

We extracted WOMAC total scores at 6 and 12 months for paired meta-analysis. The results of 6 months of follow-up showed that MSCs (including bone marrow, fat, and umbilical cord MSCs) and control groups were (the BMMSC vs. the HA, Saline, PRP, and CT: mean difference (MD) = −0.66, 95% confidence interval (CI) −0.94 to −0.38, *P*  < 0.001; MD = −3.44, 95% CI −4.4 to −2.48, *P*  < 0.001; MD = −0.11, 95% CI −0.66 to 0.45, *P*=0.704; MD = −1.5, 95% CI−2.13 to −0.883, *P*  < 0.001. The ADMSC vs. the HA, Saline and CT: MD = −0.18, 95% CI −0.64 to 0.27, *P*=0.43; MD = −1.17, 95% CI –3.08 to 0.73, *P*=0.226; MD = −1.66, 95% CI−2.72 to −0.6, *P*=0.002. The UCMSC vs. the HA: MD = −1.13, 95% CI −2.54 to 0.27, *P*=0.114).

The results of 12 months of follow-up showed that the MSCs (including bone marrow, fat, and umbilical cord MSCs) and the control group were (the BMMSC vs. the HA and PRP: MD = −1.00, 95% CI −1.41 to −0.58, *P*  < 0.001; MD = 0.04, 95% CI −0.51 to 0.60, *P*=0.879. The ADMSC vs. the HA, Saline and CT: MD = −0.25, 95% CI −0.71 to 0.21, *P*=0.218; MD = −2.51, 95% CI –3.40 to −1.62, *P*  < 0.001; MD = −2.20, 95% CI3.36 to −1.03, *P*  < 0.001. The UCMSC vs. the HA: MD = −0.03, 95% CI −0.93 to 0.88, *P*=0.956). In comparison to the control group, MSCs improved function more clearly in KOA patients.

The network meta-analysis also examined the efficacy of different MSC sources, getting WOMAC total, WOMAC stiffness, and WOMAC function scores for short-term efficacy (6 months) and long-term efficacy (12 months), respectively.

#### 3.4.2. WOMAC Total

**Figure 3:**
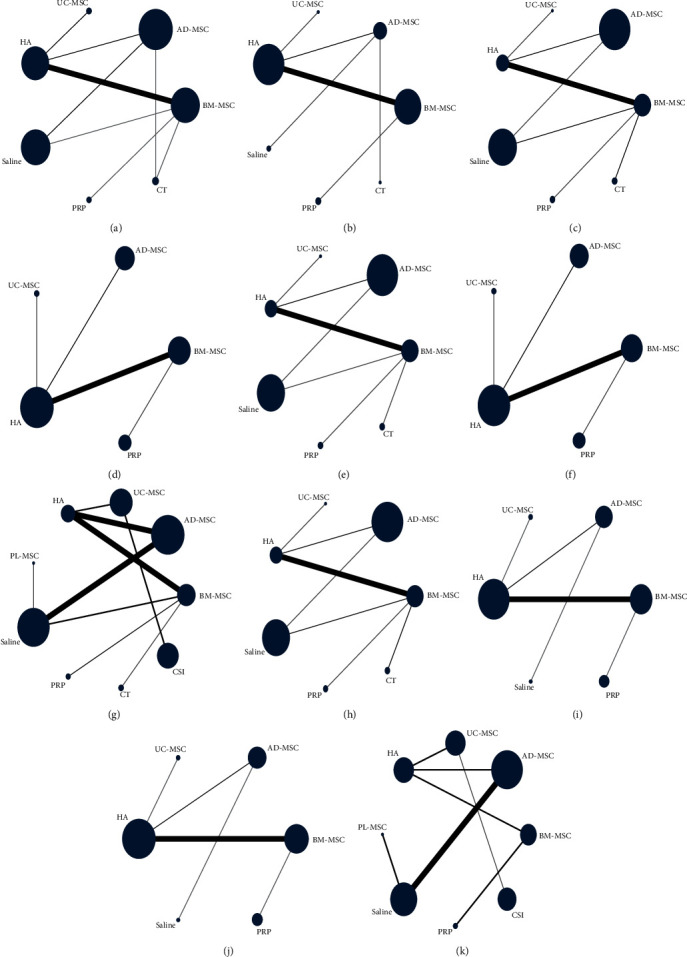
Study network geometry: (a) 6 months of WOMAC total; (b) 12 months of WOMAC total; (c) 6 months of WOMAC stiffness; (d) 12 months of WOMAC stiffness; (e) 6 months of WOMAC function; (f) 12 months of WOMAC function; (g) 6 months of VAS score; (h) 12 months of VAS score; (i) 6 months of WOMAC pain; (j) 12 months of WOMAC pain; (k) adverse event. WOMAC, Western Ontario and McMaster Universities Arthritis Index; ADMSC, adipose mesenchymal stem cell; BMMSC, bone marrow mesenchymal stem cell; UBMSC, umbilical cord mesenchymal stem cell; CT, conservative treatment; HA, hyaluronic acid; PRP, platelet-rich plasma; CSI, corticosteroid.

**Figure 4:**
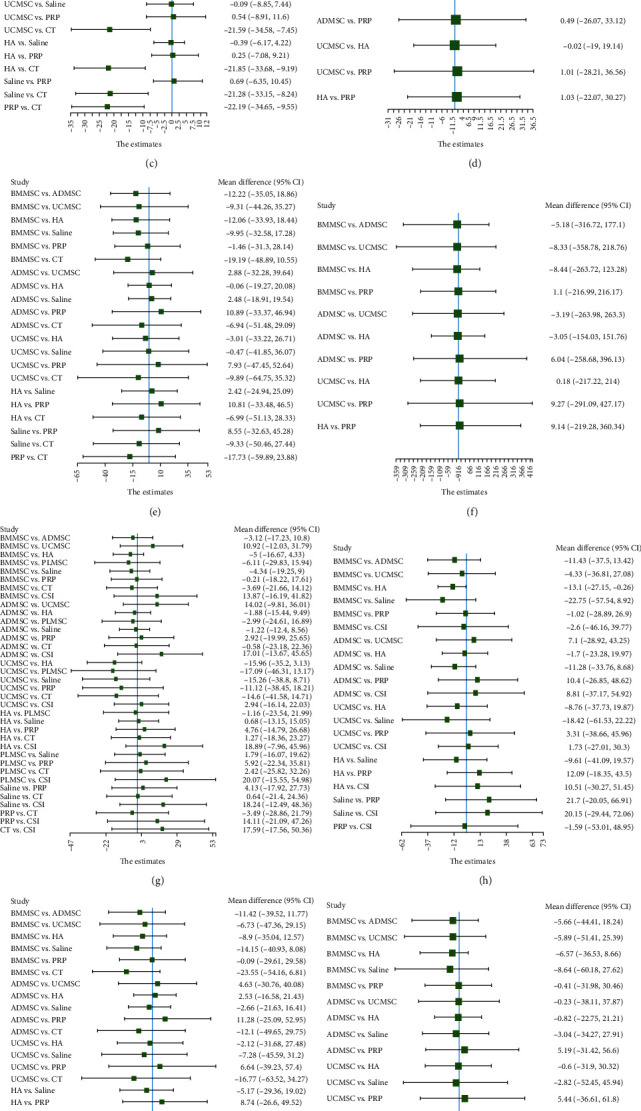
Direct comparison forest plots of the two interventions: (a) 6 months of WOMAC total; (b) 12 months of WOMAC total; (c) 6 months of WOMAC stiffness; (d) 12 months of WOMAC stiffness; (e) 6 months of WOMAC function; (f) 12 months of WOMAC function; (g) 6 months of VAS score; (h) 12 months of VAS score; (i) 6 months of WOMAC pain; (j) 12 months of WOMAC pain; (k) adverse event. MD, mean difference.

**Figure 5:**
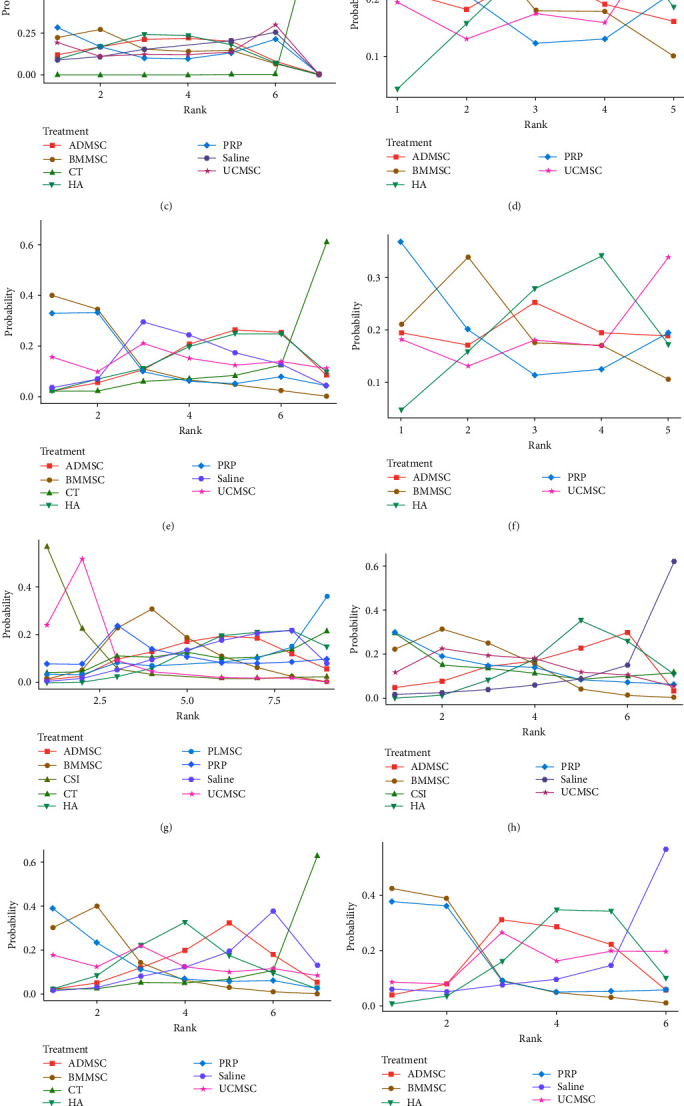
The Bayesian model is used to rank probability: (a) 6 months of WOMAC total; (b) 12 months of WOMAC total; (c) 6 months of WOMAC stiffness; (d) 12 months of WOMAC stiffness; (e) 6 months of WOMAC function; (f) 12 months of WOMAC function; (g) 6 months of VAS score; (h) 12 months of VAS score; (i) 6 months of WOMAC pain; (j) 12 months of WOMAC pain; (k) adverse event.

**Figure 6:**
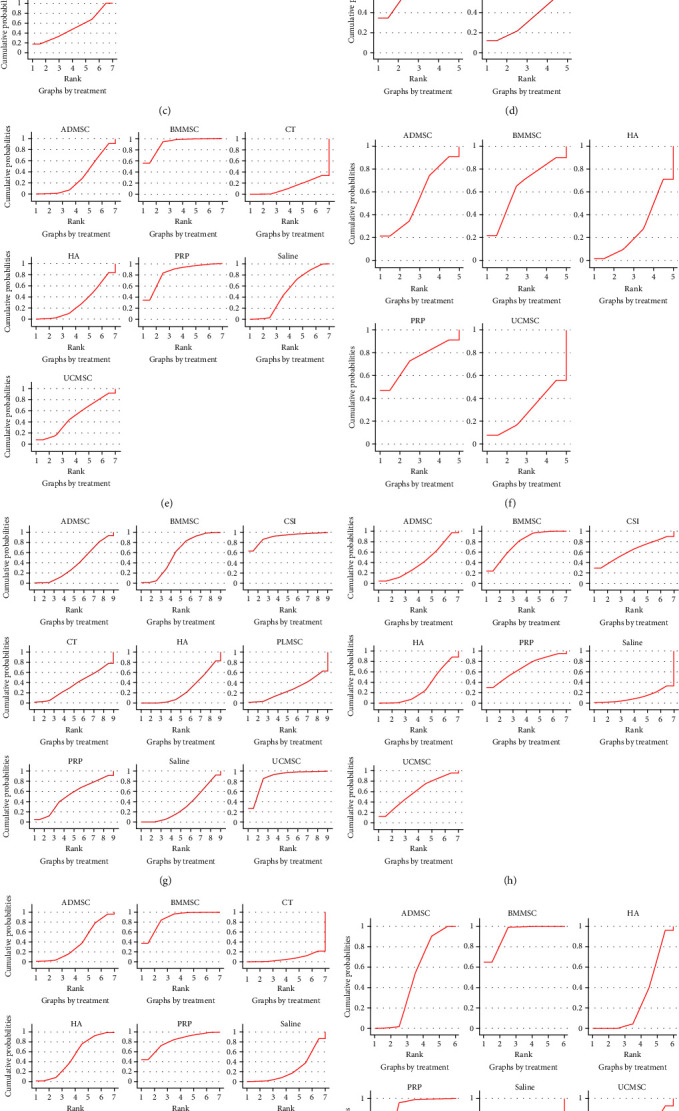
Ranking based on area under the SUCRA curve: (a) 6 months of WOMAC total; (b) 12 months of WOMAC total; (c) 6 months of WOMAC stiffness; (d) 12 months of WOMAC stiffness; (e) 6 months of WOMAC function; (f) 12 months of WOMAC function; (g) 6 months of VAS score; (h) 12 months of VAS score; (i) 6 months of WOMAC pain; (j) 12 months of WOMAC pain; (k) adverse event.

A total of 15 RCTs [19, 20, 22, 23, 26, 27, 28, 29, 30, 31, 32, 33, 34, 35, 36] at 6 months of follow-up included 813 patients reporting WOMAC total scores between MSCs of different origins (including bone marrow, fat, and umbilical cord) and the control group (Figure 3(a)). The results of the network meta-analysis, categorized by treatment modality, are presented in *Supplementary 2*. Although the analysis revealed no significant differences among the treatment modalities, the efficacy of BMMSC was superior to that of ADMSC and UCMSC (BMMSC vs. the Saline (MD = −37.64, 95% CI −143.18 to 35.37), PRP (MD = −1.72, 95% CI −129.05 to 126.49), HA (MD = −33.79, 95% CI −138.54 to 11.47), CT (MD = −28.32, 95% CI −138.25 to 60.49), UCMSC (MD = −7.81, 95% CI −158.13 to 74.99), ADMSC (MD = −20.12, 95% CI −125.24 to 42.88)) (Table 2) (Figure 4(a)). The Bayesian model ranks the methods described in the paragraph as follows: PRP, BMMSC, UCMSC, ADMSC, HA, CT, and Saline (Figure 5(a)). In addition, the ranking of areas under the SUCRA curve is UCMSC, BMMSC, PRP, ADMSC, HA, CT, and Saline (Figure 6(a)) (*Supplementary 2*).

A total of 11 RCTs [19, 21, 22, 23, 27, 29, 30, 32, 33, 34, 35] at 12 months of follow-up included 424 patients who reported WOMAC total scores between MSCs of different origins (including bone marrow, fat, and umbilical cord) and the control group (Figure 3(b)). The results of the network meta-analysis, categorized by treatment modality, are presented in *Supplementary 2*. The results showed that there was no significant difference between MSCS derived from different sources, but BMMSC had advantages over ADMSC and UCMSC (BMMSC vs. the PRP (MD = 0.98, 95% CI −670.6 to 669.71), HA (MD = −181.71, 95% CI −511.62 to 117.71), CT (MD = −200.38, 95% CI −1,090.12 to 658.64), ADMSC (MD = −176.77, 95% CI −757.1 to 378.25), UCMSC (MD = −181.55, 95% CI −937.83 to 541.13)) (Table 2) (Figure 4(b)). The Bayesian model ranks the methods described in the paragraph as follows: PRP, BMMSC, UCMSC, HA, ADMSC, CT, and Saline (Figure 5(b)). In addition, the ranking of areas under the SUCRA curve is PRP, BMMSC, ADMSC, UCMMSC, HA, CT, and Saline (Figure 6(b)) (Table S2).

#### 3.4.3. WOMAC Stiffness

A total of 10 RCTs [19, 22, 23, 26, 28, 29, 31, 32, 34, 37] at 6 months of follow-up included 543 patients reporting WOMAC stiffness scores between MSCs of different origin (including bone marrow, fat, umbilical cord) and the control group (Figure 3(c)). The findings of the network meta-analysis, organized by treatment modality, are outlined in *Supplementary 2*. Each treatment modality showed significant differences compared to CT, however, there were no statistically significant variations observed among MSCs sourced from different origins. Nevertheless, BMMSCs may possess advantages over ADMSCs and UCMSCs ((BMMSC vs. the Saline, PRP, HA, CT, ADMSC and UCMSC: MD = −0.94, 95% CI −7.87 to 3.58; MD = −0.2, 95% CI −6.34, to 6.01; MD = −0.48, 95% CI −6.55 to 4.14; MD = −22.4, 95% CI −33.5 to −11.14; MD = −0.51, 95% CI −7.27 to 4.29; MD = −0.75, 95% CI −9.74 to 6.63)) (Table 2) (Figure 4(c)). The Bayesian model ranks the methods described in the paragraph as follows: PRP, BMMSC, ADMSC, HA, Saline, UCMSC, and CT (Figure 5(c)). In addition, the ranking of areas under the SUCRA curve is BMMSC, ADMSC, PRP, HA, UCMSC, Saline, CT (Figure 6(c)) (*Supplementary 2*).

A total of six RCTs [19, 22, 23, 29, 32, 34] at 12 months of follow-up included 185 patients reporting WOMAC stiffness scores between MSCs of different origin (including bone marrow, fat, umbilical cord) and the control group (Figure 3(d)). The results of the network meta-analysis, categorized by treatment modality, are presented in *Supplementary 2*. Although the differences between treatment modalities did not reach statistical significance, BMMSCs may exhibit a slight advantage over other sources of MSCs (BMMSC vs. the PRP (MD = 0, 95% CI −19.29, to 18.99), HA (MD = −1.06, 95% CI −22.3 to 13.51), ADMSC (MD = −0.5, 95% CI −26.05 to 18.61), UCMSC (MD = −1.03, 95% CI −30.44 to 21.69)) (Table 2) (Figure 4(d)). The Bayesian model ranks the methods described in the paragraph as follows: PRP, BMMSC, ADMSC, HA, and UCMSC (Figure 5(d)). In addition, the ranking of areas under the SUCRA curve is BMMSC, PRP, ADMSC, UCMSC, and HA (Figure 6(d)) (*Supplementary 2*).

#### 3.4.4. WOMAC Functional Limitation

A total of 10 RCTs [19, 22, 23, 26, 28, 29, 31, 32, 34, 37] at 6 months of follow-up included 543 patients reporting WOMAC Functional Limitation scores between MSCs of different origin (including bone marrow, fat, umbilical cord) and the control group (Figure 3(e)). The findings of the network meta-analysis, organized by treatment modality, are outlined in *Supplementary 2*. There was no significant difference between the treatment modalities, but BMMSC may have advantages over ADMSC and UCMSC (BMMSC vs. the Saline (MD = −9.95, 95% CI −32.58 to 17.28), PRP (MD = −1.46, 95% CI −31.3, to 28.14), HA (MD = −12.06, 95% CI −33.93 to 18.44), CT (MD = −19.19, 95% CI −48.89 to 10.55), ADMSC (MD = −12.22, 95% CI −35.05 to 18.86), UCMSC (MD = −9.31, 95% CI −44.26 to 35.27)) (Table 2) (Figure 4(e)) The Bayesian model ranks the methods described in the paragraph as follows: BMMSC, PRP, UCMSC, Saline, ADMSC, HA and CT (Figure 5(e)). In addition, the ranking of areas under the SUCRA curve is BMMSC, PRP, Saline, UCMSC, ADMSC, HA, CT (Figure 6(e)) (*Supplementary 2*).

A total of six RCTs [19, 22, 23, 29, 32, 34] at 12 months of follow-up included 185 patients reporting WOMAC Functional Limitation scores between MSCs of different origin (including bone marrow, fat, umbilical cord) and the control group (Figure 3(f)). The findings of the network meta-analysis, organized by treatment modality, are outlined in *Supplementary 2*. The results showed no significant difference between each treatment modality, but BMMSC may have advantages over other sources of MSCs (BMMSC vs. the PRP (MD = 1.1, 95% CI−216.99, to 216.17), HA (MD = −8.44, 95% CI−263.72 to 123.28), ADMSC (MD = −5.18, 95% CI−316.72 to 177.1), UCMSC (MD = −8.33, 95% CI−358.78 to 218.76)) (Table 2) (Figure 4(f)). The Bayesian model ranks the methods described in the paragraph as follows: PRP, BMMSC, ADMSC, HA, and UCMSC (Figure 5(f)). In addition, the ranking of areas under the SUCRA curve is PRP, BMMSC, ADMSC, UCMSC, and HA (Figure 6(f)) (*Supplementary 2*).

#### 3.4.5. Easement of Pain

We extracted VAS scores at 6 and 12 months for paired meta-analysis. The results of 6 months of follow-up showed that the MSCs (including bone marrow, fat, umbilical cord, and placental MSCs) and the control group were (BMMSC vs. the HA, Saline, PRP, and CT : MD = −1.00, 95% CI −1.54 to −0.46, *P*  < 0.001; MD = −0.53, 95% CI −1.14 to 0.09, *P*=0.092; MD = −0.08, 95% CI −0.63 to 0.48, *P*=0.789; MD = −1.92, 95% CI−2.59 to −1.26, *P*  < 0.001. ADMSC vs. the HA, Saline: MD = −0.43, 95% CI −0.9 to 0.03, *P*=0.066; MD = −0.88, 95% CI −2.66 to 0.91, *P*=0.34. UCMSC vs. the HA and CSI: MD = −0.25, 95% CI −0.71 to 0.21, *P*=0.218; MD = 0.11, 95% CI − 0.15 to 0.36, *P*=0.42. PLMSC vs. the Saline: MD = 1.75, 95% CI 0.7 to 2.79, *P*=0.001).

The results of 12 months of follow-up showed that the MSCs (including bone marrow, fat, umbilical cord MSCs) and the control group were (BMMSC vs. the HA and PRP: MD = −1.18, 95% CI −1.52 to −0.84, *P*=0.001; MD = −0.43, 95% CI − 0.99 to 0.14, *P*=0.137. ADMSC vs. the HA and Saline: MD = −0.43, 95% CI −0.89 to 0.04, *P*=0.071; MD = −2.22, 95% CI −3.38 to −1.02, *P*=0.001. UCMSC vs. the HA and CSI: MD = −0.97, 95% CI −1.93 to −0.01, *P*=0.047; MD = 0.058, 95% CI –0.20 to 0.312, *P*=0.656). Compared with the control group, MSCs had more obvious effects on pain relief in KOA patients.

The network meta-analysis evaluated the efficacy of different MSC sources, extracting VAS and WOMAC pain ratings for short-term (6 months) and long-term (12 months) efficacy.

#### 3.4.6. VAS Score

A total of 14 RCTs [19, 22, 23, 26, 28, 29, 31, 32, 33, 34, 35, 37, 38, 39] at 6 months of follow-up included 858 patients who reported VAS scores between different sources of MSCs (including bone marrow, fat, umbilical cord, placenta) and control subjects (Figure 3(g)). The results of the network meta-analysis, categorized by treatment modality, are presented in *Supplementary 2*. The results indicated no statistically significant variance among the different treatment modalities, however UCMSC may present potential advantages over other sources of MSCs (UCMSC vs. the Saline (MD = −15.26, 95% CI −38.8 to 8.71), PRP (MD = −11.12, 95% CI −38.45 to 18.21), HA (MD = −15.96, 95% CI −35.2 to 3.13), CT (MD = −14.6, 95% CI −41.58 to 14.71), BMMSC (MD = −10.92, 95% CI −31.79 to 12.03), ADMSC (MD = −14.02, 95% CI −36.01 to 9.81), PLMSC (MD = −17.09, 95% CI −46.31 to 13.17), CSI (MD = 2.94, 95% CI −16.14 to 22.03)) (Table 3) (Figure 4(g)). The Bayesian model ranks the methods described in the paragraph as follows: CSI, UCMSC, PRP, BMMSC, ADMSC, HA, Saline, CT, and PLMSC (Figure 5(g)). In addition, the ranking of areas under the SUCRA curve is CSI, UCMSC, BMMSC, PRP, ADMSC, CT, Saline, PLMSC, and HA (Figure 6(g)) (*Supplementary 2*).

A total of 12 RCTs [19, 21, 22, 23, 29, 30, 32, 33, 34, 35, 37, 39] at 12 months of follow-up included 655 patients reporting VAS scores between different sources of MSCs (including bone marrow, fat, umbilical cord, and placenta) and controls (Figure 3(h)). The results of the network meta-analysis, categorized by treatment modality, are presented in *Supplementary 2*. The findings revealed no significant variance among the different treatment modalities, although BMMSCs may offer potential advantages over other sources of MSCs (BMMSC vs. the Saline (MD = −22.75, 95% CI −57.54 to –−8.92), PRP (MD = −1.02, 95% CI −28.89 to 26.9), HA (MD = −13.1, 95% CI −27.15 to −0.26), UCMSC (MD = −4.33, 95% CI −36.81 to 27.08), ADMSC (MD = −11.43, 95% CI −37.5 to 13.42), CSI (MD = −2.6, 95% CI −46.16 to 39.77)) (Table 3) (Figure 4(h)) The Bayesian model ranks the methods described in the paragraph as follows: PRP, BMMSC, CSI, UCMSC, HA, ADMSC, and Saline (Figure 5(h)). In addition, the ranking of areas under the SUCRA curve is BMMSC, PRP, CSI, UCMSC, ADMSC, HA, and Saline (Figure 6(h)) (*Supplementary 2*).

#### 3.4.7. WOMAC Pain Score

A total of 10 RCTs [19, 22, 23, 26, 28, 29, 31, 32, 34, 37] at 6 months of follow-up included 544 patients reporting WOMAC pain scores between MSCs of different origin (including bone marrow, fat, umbilical cord) and the control group (Figure 3(i)). The results of the network meta-analysis, categorized by treatment modality, are presented in *Supplementary 2*. The results showed that the differences between the treatment modalities were not statistically significant, but BMMSC may have advantages over other sources of MSCs (BMMSC vs. the Saline (MD = −14.15, 95% CI −40.93 to 8.08), PRP (MD = −0.09, 95% CI −29.61 to 29.58), HA (MD = −8.9, 95% CI −35.04 to 12.57), CT (MD = −23.55, 95% CI −54.16 to 6.81), ADMSC (MD = −11.42, 95% CI −39.52 to 11.77), UCMSC (MD = −6.73, 95% CI −47.36 to 29.15)) (Table 3) (Figure 4(i)). The Bayesian model ranks the methods described in the paragraph as follows: PRP, BMMSC, UCMSC, HA, ADMSC, Saline, and CT (Figure 5(i)). In addition, the ranking of areas under the SUCRA curve is BMMSC, PRP, UCMSC, HA, ADMSC, Saline, CT (Figure 6(i)) (*Supplementary 2*).

A total of eight RCTs [19, 21, 22, 23, 29, 32, 34, 37] at 12 months of follow-up included 228 patients reporting WOMAC pain scores between MSCs of different origin (including bone marrow, fat, umbilical cord) and the control group (Figure 3(j)). The results of the network meta-analysis, categorized by treatment modality, are presented in *Supplementary 2*. The differences between the treatments were not statistically significant, but BMMSC may have advantages over other sources of MSCs (BMMSC vs. the Saline (MD = −8.64, 95% CI−60.18 to 27.62), PRP (MD = −0.41, 95% CI−31.98, to 30.46), HA (MD = −6.57, 95% CI−36.53 to 8.66), ADMSC (MD = −5.66, 95% CI−44.41 to 18.24), UCMSC (MD = −5.89, 95% CI−51.41 to 25.39)) (Table 3) (Figure 4(j)). The Bayesian model ranks the methods described in the paragraph as follows: BMMSC, PRP, ADMSC, UCMSC, HA, and Saline (Figure 5(j)). Furthermore, the following regions are ranked under the SUCRA curves: BMMSC, PRP, ADMSC, UCMSC, HA, and Saline (Figure 6(j)) (*Supplementary 2*).

#### 3.4.8. Safety

A total of 12 RCTs involving 894 patients documented adverse events, such as joint swelling, joint pain, and synovitis, following intra-articular injection (Figure 3(k)). The findings indicated that PLMSCs exhibited a higher incidence of adverse events compared to other sources of MSCs, possibly attributed to the limited number of studies on PLMSCs (BMMSC vs. the PLMSC, ADMSC and UCMSC: MD = −31.85, 95% CI −101.21 to −3.3; MD = −0.86, 95% CI −3.7, to 1.63; MD = −0.11, 95% CI −3.61 to 3.24) (Table 3) (Figure 4(k)). While there were no statistically significant differences among BMMSCs, ASDMSCs, and UCMSCs in terms of safety profile, BMMSCs may have a potential advantage in this aspect. The Bayesian model ranks the methods described in the paragraph as follows: PRP, CSI, UCMSC, HA, BMMSC, Saline, ADMSC, and PLMSC (Figure 5(k)). Furthermore, the following regions are ranked under the SUCRA curves: PRP, CSI, HA, BMMSC, UCMSC, Saline, ADMSC, and PLMSC (Figure 6(k)) (*Supplementary 2*).

### 3.5. Sensitivity Analysis and Publication Bias

Publication bias was evaluated using Egger's test for WOMAC, VAS, and AE, which showed no bias. Heterogeneity was significantly reduced after removing the study by Kim et al. [31], by excluding the included studies one by one using sensitivity analysis. However, the Kim et al. [31] study did not affect the pooled results, which were stable.

### 3.6. GRADE Quality Evaluation

The key outcome indicators (WOMAC, VAS, AE) from the included studies were assessed using the GRADE score index, which evaluates five factors: risk of bias, inconsistency, imprecision, indirectness, and publication bias. The quality of evidence was categorized into four levels: high, moderate, low, and very poor. According to the GRADE scoring system, WOMAC, VAS, and AE scores are low-quality scores.

## 4. Discussion

OA is a common disease globally that negatively impacts individuals' quality of life. The main treatment approaches for OA include conservative treatment and surgical interventions such as total knee arthroplasty and total hip arthroplasty [40]. Surgical treatment can partially relieve joint deformities and pain in patients with end-stage OA; however, the surgical risks and associated complications cannot be overlooked. This is particularly important for elderly patients with a higher risk of complications, such as infections, thrombosis, and the need for secondary surgeries [41].

For conservative treatment, pharmacological therapies, especially NSAIDs, are widely used in clinical practice. However, their primary role is pain control with no reparative effects on cartilage [42]. Therefore, cell transplantation has attracted considerable attention as a novel therapeutic approach. Among them, MSCs have emerged as a popular choice for treating OA because of their wide availability (from bone marrow, adipose tissue, muscle, periosteum, and synovium), ease of accessibility, safety, and potential to differentiate into cartilage [43, 44, 45]. These have demonstrated promising results in clinical settings.

In an RCT, Lee et al. [20] treated 12 patients with OA by intra-articular injection of autologous ADMSCs. The MSCs maintained the morphological changes in the cartilage and effectively relieved pain in patients. In a study by Matas et al. [23], UCMSCs were injected intra-articularly, and patients were followed up for 12 months. Clinical scores and magnetic resonance imaging findings were compared, and the results revealed significant improvements in pain and function, with no evidence of cartilage damage on magnetic resonance imaging. Moreover, Lamo-Espinosa et al. [33] injected different doses of BMMSCs into the joint cavity of patients with OA, resulting in reduced pain and improved functional activities. In a four-arm RCT, researchers used autologous bone marrow aspirate concentrate, umbilical cord MSCs, autologous adipose stromal vascular fraction, and corticosteroids in a comparison, and the results showed that umbilical cord MSCs did not have a clear advantage [39]. MSCs from various sources have demonstrated significant clinical efficacy in OA treatment, improving pain relief and functional activities in patients with OA. However, differences have not been reported in the therapeutic efficacy of different types of MSCs for OA.

Therefore, we discussed the comparative efficacy of different sources of MSCs (including MSMSCs, ADMSCs, PLMSCs, and UCMSCs) and control groups in treating KOA. Traditional and network meta-analyses were conducted based on a Bayesian framework. The results indicated good heterogeneity between the MSC and control groups. Regarding functional improvement, MSCs from different sources demonstrated improvement in the WOMAC total score for patients with OA compared to the PRP and HA control groups; however, the results were not significant. Numerous studies have demonstrated the clinical efficacy of PRP for KOA. However, the results vary owing to the differences in the preparation process. For example, Park et al. [46] conducted a randomized trial involving patients with KOA who received intra-articular injections of PRP or HA. PRP improved both function and pain, with PRP rich in high concentrations of growth factors demonstrating better efficacy than the other groups. However, some studies have revealed that PRP does not improve ankle OA [47]. Overall, PRP and HA have good clinical effects on KOA. MSCs from various sources revealed significant differences compared with the conservative treatment or Saline control groups, demonstrating significant improvement. Moreover, different stem cell sources improved significantly in the long-term follow-up than the no-cell therapy control group. A further reticulation meta-analysis showed that the differences between all treatment modalities were not statistically significant in the WOMAC score metrics at 6 and 12 months of follow-up, and since all treatment modalities can improve patients' symptoms clinically, we observed the reticulation meta-analysis results to find that BMMSC may be advantageous among MSCs of different origins. In addition, in the WOMAC subscales (including WOMAC stiffness and WOMAC functional limitation), although only the WOMAC stiffness rating scale was statistically significant for each treatment modality compared to CT, observing the results of the reticulation meta-analysis also revealed that BMMSC may be advantageous among different sources of MSCs. of MSCs. The advantage of BMMSCs in functional improvement has not been confirmed in previous meta-analyses; however, multiple clinical studies have demonstrated their treatment advantages [21, 33]. This may be attributed to the inherent self-renewal ability of BMMSCs and the potential for growth factor production and differentiation into chondrocytes, adipocytes, and osteoblasts [15, 48]. Regarding pain relief, traditional meta-analyses demonstrated good heterogeneity, indicating that MSCs from different sources have significant analgesic effects in patients with OA. Further network meta-analysis of different sources of MSCs showed that only the results of the differences between BMMSC and HA were statistically significant in terms of improvement in VAS scores at 6 and 12 months of follow-up and that the differences between the other different treatment modalities were not statistically significant. However, in the results of the network meta-analysis, it was found that BMMSC may still be advantageous in different MSCs. Also, in the WOMAC pain score, although the differences between treatment modalities were not statistically significant, BMMSC and UCMSC may still have an advantage over ADMSC. A meta-analysis also demonstrated that UCMSC was advantageous in pain relief in comparisons involving various doses [49]. Additionally, in a clinical study, repeated injections were more effective than single injections [23]. These studies indicate that injection dosage may also improve pain in patients with KOA, although no recommended dosage has been established. MSCs significantly inhibit bone loss in the posterior medial femur area in patients with KOA after intra-articular injection of synovial MSCs [50]. This indicates that the reparative effect of MSCs on cartilage may be the key to improving function and relieving pain in patients with OA. Conventional treatments such as physical therapy, NSAIDs, and surgery can eliminate inflammation, swelling, joint adhesion, and stiffness but do not improve cartilage damage [51]. In the phase I/IIa trial, Chahal et al. [52] injected BMMSCs into the knee joint at different concentrations and discovered a significant decrease in cartilage degradation biomarkers and magnetic resonance imaging synovitis. Furthermore, Zhang et al. [53] demonstrated that UCMSCs downregulated the expression of matrix metalloproteinase-5 and upregulated the expression of type II collagen and Ki67 in articular cartilage in an iodine-acetic acid-induced OA rat model. Additionally, ADMSCs alleviated inflammatory reactions and cartilage degeneration in an OA mouse model and increased glycosaminoglycan production, inducing endogenous cartilage formation. In summary, studies have shown that MSCs play an important role in cartilage regeneration and repair, but the injection of MSCs in the clinic faces many problems, mainly including the lack of a strict standard for the injection dose. Recently, a study conducted a net meta-analysis on the injection dose of MSCs, and the results showed that the clinical efficacy of the high-dose group was better than that of the low-dose group, but there was an increase in the number of adverse events, which is also a real problem [54]. For the safety of MSCs, we performed a network meta-analysis of the adverse events associated with the injections, and the results showed that the safety of PLMSCs differed significantly from other treatment modalities, but only one study was included for PLMSCs, so more clinical studies may be needed to further confirm the safety of PLMSCs. There was no statistically significant difference in AE between different sources of MSCs, suggesting that there may be no difference in safety compared to other therapeutic modalities or between different sources of MSCs, and therefore, safety should be recognized. Overall, our study conducted a meta-analysis of MSCs from various sources. Although the comparisons among the included MSC studies were indirect, which could be considered a limitation, we systematically compared long-term and short-term outcome metrics based on the follow-up duration. We designated 6 months as the timeframe for short-term efficacy evaluation and 12 months for long-term efficacy evaluation. This suggests that future efficacy studies should include longer follow-up periods. Our study had some limitations. MSCs play a crucial role in cartilage regeneration and repair. However, owing to the lack of quantitative evaluation methods for cartilage assessment, we did not analyze the cartilage. Second, the lack of clinical research on synovial MSCs and other clinical studies is another reason they were not included in this study. Lastly, no recommended dosage has been established for MSC injections in different studies; thus, we used the lowest dosage in this study. More clinical trials are needed to evaluate the dosage, clinical efficacy, and safety of MSCs from other sources in patients with OA. Therefore, more in-depth research is needed for the clinical application of MSCs, not only in terms of clinical efficacy but also in terms of the ethical issues involved in the collection of cells, which may include the informed consent of the donor, the distribution of benefits in the case of commercial utilization, the fairness of the subjects, and the privacy of the subjects.

## 5. Conclusion

In conclusion, we used a network meta-analysis to evaluate the short-term and long-term efficacy differences between different sources of MSCs (ADMSCs, BMMSCs, and UCMSCs) in patients with KOA. Our findings suggest that the traditional meta-analysis showed that MSCS from different sources were able to improve KOA compared to the control group. However, the difference in efficacy between the various MSCS was not significant in the network meta-analysis. Compared with MSCs from other sources, BMMSCs may improve the short-term and long-term knee function of KOA patients more significantly, while UCMSCs and ADMSCs are relatively weak. In addition, UCMSCs and BMMSCs may be superior to ADMSCs in short-term and long-term pain relief in KOA patients. Our findings demonstrate the potential benefits of MSCs, which will lead to better recommendations for clinical and scientific research in the treatment of OA and other diseases with MSCs and may provide good suggestions for more in-depth research on MSCs in the future. However, owing to the limitations of this study, more accurate efficacy differences between MSCs from different sources, larger clinical samples, and long-term follow-up data are required.

## Figures and Tables

**Table 1:** Basic information of the included studies.

Study	Year	Trial number	Sample size	Intervention	KOA severity	Cell source	Mean age	Sex (M : F)	Outcomes	Follow-up
Aurelio Vega	2015	NCT01586312	BMMSC = 15 HA = 15	40 × 10^6^	KLⅡ–Ⅳ	Allogeneic	57 ± 9	13 : 17	WOMAC, VAS	12 m
Chen-FongChen	2021	NCT02784964	ADMSC = 17 HA = 8	16 × 10^6^	KLⅡ–Ⅲ	Allogeneic	67.6 ± 6.60	11 : 46	WOMAC, VAS	6 m, 12 m
Jose Matas	2019	NCT02580695	UCMSC = 10 HA = 9	20 × 10^6^	KLⅡ–Ⅲ	Allogeneic	55.48 ± 5.7	8 : 11	WOMAC, VAS	6 m, 12 m
Liang Jing Lu	2019	NCT02162693	ADMSC = 26 HA = 26	5 × 10^7^	KLⅠ–Ⅲ	Autologous	57.33 ± 8.02	6 : 46	WOMAC, VAS	6 m, 12 m
Kevin	2022	CUHK_CCT00469	BMMSC = 10 HA = 10	1 × 10^6^	KLⅡ–Ⅲ	Autologous	57.9 ± 4.5	8 : 12	WOMAC, VAS	6 m, 12 m
José María	2016	NCT02123368	BMMSC = 10 HA = 10	10 × 10^6^	KLⅡ–Ⅳ	Autologous	63.09 ± 6.24	13 : 04	WOMAC, VAS	6 m
Mohsen	2018	NCT01504464	BMMSC = 19 Saline = 24	40 × 10^6^	KLⅡ–Ⅳ	Autologous	53.37 ± 7.34	27 : 16	WOMAC, VAS	6 m
Woo-Suk Lee	2019	NCT02658344	ADMSC = 12 Saline = 12	1 × 10^8^	KLⅡ–Ⅳ	Autologous	62.7 ± 10.81	6 : 18	WOMAC	6 m
Shayesteh	2018	IRCT2015101823298N	PLMSC = 10 Saline = 10	(0.5−0.6) × 10^8^	KLⅡ–Ⅳ	Allogeneic	NA	NA	VAS	6 m, 12 m
Julien Freitag	2019	ACTRN12614000814673	ADMSC = 9 CT = 10	100 × 10^6^	KLⅡ–Ⅲ	Autologous	53.05 ± 6.24	12 : 8	WOMAC	6 m, 12 m
Gupta	2016	NCT01453738	BMMSC = 10 HA = 10	25 × 10^6^	KLⅡ–Ⅲ	Allogeneic	56.5 ± 7.31	3 : 17	WOMAC, VAS	6 m, 12 m
D. Kuah	2018	ACTRN12615000439549	ADMSC = 8 Saline = 4	3.9 × 10^7^	KLⅠ–Ⅲ	Allogeneic	52.2 ± 8.22	7 : 5	WOMAC, VAS	6 m, 12 m
Lamo-Espinosa	2020	NCT02365142	BMMSC = 24 PRP = 26	100 × 10^6^	KLⅡ–Ⅲ	Autologous	55.27 ± 36.8	33 : 17	WOMAC, VAS	6 m, 12 m
Wang, Y	2016	NA	UCMSC = 18 HA = 18	(2–3) × 107	NA	Allogeneic	53.32 ± 0.96	21 : 15	WOMAC	6 m
Mendoza	2018	NCT01485198	BMMSC = 26 CT = 25	20 × 10^6^	KLⅡ–Ⅲ	Autologous	57.52 ± 11.49	16 : 45	WOMAC, VAS	6 m
Bahareh	2023	IRCT20080728001031N23	ADMSC = 20 Saline = 20	100 × 10^6^	KLⅡ–Ⅲ	Allogeneic	54.48 ± 7.32	4 : 36	WOMAC, VAS	6 m, 12 m
Kang-Il Kim	2023	NCT03990805	ADMSC = 125 Saline = 127	1 × 108	KLⅢ	Autologous	63.75 ± 7.08	65 : 187	WOMAC, VAS	6 m
Ken	2023	NCT03818737	UCMSC = 118 HA = 120	NA	KLⅡ–Ⅳ	Allogeneic	58.1 ± 8.13	102 : 136	VAS	6 m, 12 m
Pawan	2023	CTRI/2018/09/015785	BMMSC = 73 HA = 73	25 × 10^6^	KLⅡ–Ⅲ	Allogeneic	52.6 ± 6.83	48 : 98	WOMAC, VAS	6 m, 12 m

Abbreviations: ADMSC, adipose-derived mesenchymal stem cell; BMMSC, bone marrow mesenchymal stem cell; UBMSC, umbilical-derived mesenchymal stem cell; HA, hyaluronic acid; PLMSC, placenta-derived mesenchymal stem cells; CT, conservative treatment; PRP, platelet-rich plasma; CSI, corticosteroid; WOMAC, Western Ontario and McMaster Universities Arthritis Index; VAS, visual analog scale; KL, Kellgren and Lawrence System; NA, no answer; m, month.

**Table 2:** WOMAC total, WOMAC stiffness, and WOMAC functional paired comparison results.

	Comparison	MD (95% CI)−6 mo	Comparison	MD (95% CI)−12 mo
WOMAC total	BMMSC vs. ADMSC	MD = −20.12, 95% CI −125.24 to 42.88	BMMSC vs. ADMSC	MD = −176.77, 95% CI −757.1 to 378.25
	BMMSC vs. UCMSC	MD = −7.81, 95% CI −158.13 to 74.99	BMMSC vs. UCMSC	MD = −181.55, 95% CI −937.83 to 541.13
	BMMSC vs. HA	MD = −33.79, 95% CI −138.54 to 11.47	BMMSC vs. HA	MD = −181.71, 95% CI −511.62 to 117.71
	BMMSC vs. PRP	MD = −1.72, 95% CI −129.05 to 126.49	BMMSC vs. PRP	MD = 0.98, 95% CI −670.6 to 669.71
	BMMSC vs. Saline	MD = −37.64, 95% CI −143.18 to 35.37	BMMSC vs. CT	MD = −200.38, 95% CI −1090.12 to 658.64
	BMMSC vs. CT	MD = −28.32, 95% CI −138.25 to 60.49	—	—

WOMAC stiffness	BMMSC vs. ADMSC	MD = −0.51, 95% CI −7.27 to 4.29	BMMSC vs. ADMSC	MD = −0.5, 95% CI −26.05 to 18.61
	BMMSC vs. UCMSC	MD = −0.75, 95% CI −9.74 to 6.63	BMMSC vs. UCMSC	MD = −1.03, 95% CI −30.44 to 21.69
	BMMSC vs. HA	MD = −0.48, 95% CI −6.55 to 4.14	BMMSC vs. HA	MD = −1.06, 95% CI −22.3 to 13.51
	BMMSC vs. PRP	MD = −0.2, 95% CI −6.34, to 6.01	BMMSC vs. PRP	MD = 0, 95% CI −19.29, to 18.99
	BMMSC vs. Saline	MD = −0.94, 95% CI −7.87 to 3.58	—	—
	BMMSC vs. CT	MD = −22.4, 95% CI −33.5 to −11.14	—	—

WOMAC functional	BMMSC vs. ADMSC	MD = −12.22, 95% CI −35.05 to 18.86	BMMSC vs. ADMSC	MD = −5.18, 95% CI−316.72 to 177.1
	BMMSC vs. UCMSC	MD = −9.31, 95% CI −44.26 to 35.27	BMMSC vs. UCMSC	MD = −8.33, 95% CI−358.78 to 218.76
	BMMSC vs. HA	MD = −12.06, 95% CI −33.93 to 18.44	BMMSC vs. HA	MD = −8.44, 95% CI−263.72 to 123.28
	BMMSC vs. PRP	MD = −1.46, 95% CI −31.3, to 28.14	BMMSC vs. PRP	MD = 1.1, 95% CI−216.99, to 216.17
	BMMSC vs. Saline	MD = −9.95, 95% CI −32.58 to 17.28	—	—
	BMMSC vs. CT	MD = −19.19, 95% CI −48.89 to 10.55	—	—

Abbreviations: ADMSC, adipose-derived mesenchymal stem cell; BMMSC, bone marrow mesenchymal stem cell; UBMSC, umbilical-derived mesenchymal stem cell; HA, hyaluronic acid; PLMSC, placenta-derived mesenchymal stem cells; CT, conservative treatment; PRP, platelet-rich plasma; CSI, corticosteroid; MD, mean difference; 95% CI, 95% confidence interval; mo, month.

**Table 3:** VAS, WOMAC pain, and safety paired comparison results.

	Comparison	MD (95% CI)−6 mo	Comparison	MD (95%CI)−12 mo
VAS	UCMSC vs. BMMSC	MD = −10.92, 95% CI −31.79 to 12.03	BMMSC vs. ADMSC	MD = −11.43, 95% CI −37.5 to 13.42
	UCMSC vs. ADMSC	MD = −14.02, 95% CI −36.01 to 9.81	BMMSC vs. UCMSC	MD = −4.33, 95% CI −36.81 to 27.08
	UCMSC vs. PLMSC	MD = −17.09, 95% CI −46.31 to 13.17	BMMSC vs. HA	MD = −13.1, 95% CI −27.15 to −0.26
	UCMSC vs. HA	MD = −15.96, 95% CI −35.2 to 3.13	BMMSC vs. PRP	MD = −1.02, 95% CI −28.89 to 26.9
	UCMSC vs. PRP	MD = −11.12, 95% CI −38.45 to 18.21	BMMSC vs. CSI	MD = −2.6, 95% CI −46.16 to 39.77
	UCMSC vs. CSI	MD = 2.94, 95% CI −16.14 to 22.03	BMMSC vs. Saline	MD = −22.75, 95% CI −57.54 to −8.92
	UCMSC vs. Saline	MD = −15.26, 95% CI −38.8 to 8.71	—	—
	UCMSC vs. CT	MD = −14.6, 95% CI −41.58 to 14.71	—	—

WOMAC pain	BMMSC vs. ADMSC	MD = −11.42, 95% CI −39.52 to 11.77	BMMSC vs. ADMSC	MD = −5.66, 95% CI−44.41to 18.24
	BMMSC vs. UCMSC	MD = −6.73, 95% CI −47.36 to 29.15	BMMSC vs. UCMSC	MD = −5.89, 95% CI−51.41 to 25.39
	BMMSC vs. HA	MD = −8.9, 95% CI −35.04 to 12.57	BMMSC vs. HA	MD = −6.57, 95% CI−36.53 to 8.66
	BMMSC vs. PRP	MD = −0.09, 95% CI −29.61 to 29.58	BMMSC vs. PRP	MD = −0.41, 95% CI−31.98, to 30.46
	BMMSC vs. Saline	MD = −14.15, 95% CI −40.93 to 8.08	BMMSC vs. Saline	MD = −8.64, 95% CI−60.18 to 27.62
	BMMSC vs. CT	MD = −23.55, 95% CI −54.16 to 6.81	—	—

Safety	—	—	BMMSC vs. ADMSC	MD = −0.86, 95% CI −3.7, to 1.63
	—	—	BMMSC vs. UCMSC	MD = −0.11, 95% CI −3.61 to 3.24
	—	—	BMMSC vs. PLMSC	MD = −31.85, 95% CI −101.21 to −3.3

Abbreviations: ADMSC, adipose-derived mesenchymal stem cell; BMMSC, bone marrow mesenchymal stem cell; UBMSC, umbilical-derived mesenchymal stem cell; HA, hyaluronic acid; PLMSC, placenta-derived mesenchymal stem cells; CT, conservative treatment; PRP, platelet-rich plasma; CSI, corticosteroid; MD, mean difference; 95% CI, 95% confidence interval; mo, month.

## Data Availability

The original contributions presented in the study are included in the article/supplementary material; further inquiries can be directed to the corresponding author.

## Supplementary Materials

Supplementary 1 The supplementary file includes the Database search formula of the manuscript.

Supplementary 2 Table S1: pairwise meta-analysis for each outcome based on random effects model. Table S2: SUCRA results of interventions for each outcome. Figure S1: publication of bias evaluation results.
