# Supplementary material for: Comparative Analysis of Short-Term and Long-Term Clinical Efficacy of Mesenchymal Stem Cells from Different Sources in Knee Osteoarthritis: A Network Meta-Analysis
Source: Stem Cells Int. 2024 May 31;2024:2741681. doi: 10.1155/2024/2741681 (PMC11178400; doi:10.1155/2024/2741681)
Supplement: Supplementary 1 — The supplementary file includes the Database search formula of the manuscript. [file 2741681.f1.docx]

((("Mesenchymal Stem Cells"[Mesh]) OR(Stem,Cell,,Mesenchymal,OR,Mesenchymal,Stem,Cell,OR,Stem,Cells,,Mesenchymal,OR,Bone,Marrow,Mesenchymal,Stem,Cells,OR,Bone,Marrow,Mesenchymal,Stem,Cell,OR,Bone,Marrow,Stromal,Cells,OR,Bone,Marrow,Stromal,Cell,OR,Bone,Marrow,Stromal,Cells,,Multipotent,OR,Multipotent,Bone,Marrow,Stromal,Cell,OR,Multipotent,Bone,Marrow,Stromal,Cells,OR,Adipose-Derived,Mesenchymal,Stem,Cells,OR,Adipose,Derived,Mesenchymal,Stem,Cells,OR,Adipose-Derived,Mesenchymal,Stromal,Cells,OR,Adipose,Derived,Mesenchymal,Stromal,Cells,OR,Mesenchymal,Stem,Cells,,Adipose-Derived,OR,Mesenchymal,Stem,Cells,,Adipose,Derived,OR,Adipose-Derived,Mesenchymal,Stem,Cell,OR,Adipose,Derived,Mesenchymal,Stem,Cell,OR,Adipose,Tissue-Derived,Mesenchymal,Stem,Cell,OR,Adipose,Tissue,Derived,Mesenchymal,Stem,Cell,OR,Adipose,Tissue-Derived,Mesenchymal,Stem,Cells,OR,Adipose,Tissue,Derived,Mesenchymal,Stem,Cells,OR,Adipose,Tissue-Derived,Mesenchymal,Stromal,Cells,OR,Adipose,Tissue,Derived,Mesenchymal,Stromal,Cells,OR,Adipose,Tissue-Derived,Mesenchymal,Stromal,Cell,OR,Adipose,Tissue,Derived,Mesenchymal,Stromal,Cell,OR,Mesenchymal,Stromal,Cells,OR,Mesenchymal,Stromal,Cell,OR,Stromal,Cell,,Mesenchymal,OR,Stromal,Cells,,Mesenchymal,OR,Multipotent,Mesenchymal,Stromal,Cells,OR,Multipotent,Mesenchymal,Stromal,Cell,OR,Mesenchymal,Stromal,Cells,,Multipotent,OR,Mesenchymal,Progenitor,Cell,OR,Mesenchymal,Progenitor,Cells,OR,Progenitor,Cell,,Mesenchymal,OR,Progenitor,Cells,,Mesenchymal,OR,Wharton,Jelly,Cells,OR,Wharton's,Jelly,Cells,OR,Wharton's,Jelly,Cell,OR,Whartons,Jelly,Cells,OR,Bone,Marrow,Stromal,Stem,Cells)) AND (("Osteoarthritis"[Mesh]) OR (Osteoarthritides OR Osteoarthrosis OR Osteoarthroses OR Arthritis, Degenerative OR Arthritides, Degenerative OR Degenerative Arthritides OR Degenerative Arthritis OR Arthrosis OR Arthroses OR Osteoarthrosis Deformans OR Knee Osteoarthritides OR Knee Osteoarthritis OR Osteoarthritis of Knee OR Osteoarthritis of the Knee))) AND ((clinical[tiab] AND trial[tiab]) OR "clinical trials as topic"[mesh] OR "clinical trial"[pt] OR random*[tiab] OR "random allocation"[mesh] OR "therapeutic use"[sh])
