# Supplementary material for: Comparative Analysis of Short-Term and Long-Term Clinical Efficacy of Mesenchymal Stem Cells from Different Sources in Knee Osteoarthritis: A Network Meta-Analysis
Source: Stem Cells Int. 2024 May 31;2024:2741681. doi: 10.1155/2024/2741681 (PMC11178400; doi:10.1155/2024/2741681)
Supplement: Supplementary 2 — Table S1: pairwise meta-analysis for each outcome based on random effects model. Table S2: SUCRA results of interventions for each outcome. Figure S1: publication of bias evaluation results. [file 2741681.f2.docx]

**Supplementary Table S1. Pairwise meta-analysis for each outcome based on random effects model.**

| Six months and twelve months of WOMAC Total | | | | | | | |  |
| --- | --- | --- | --- | --- | --- | --- | --- | --- |
| ADMSC | ADMSC | -20.12(-125.24, 42.88) | 8.69 (-102.5, 97.85) | 11.84 (-50.5, 100.73) | -17.94 (-192.59, 113.25) | 17.81 (-54.72, 79.81) | -12.73(-117.06, 118.85) |  |
| BMMSC | 176.77(-378.25, 757.1) | BMMSC | 28.32 (-60.49, 138.25) | 33.79 (-11.47, 138.54) | 1.72 (-126.49, 129.05) | 37.64 (-35.37, 143.18) | 7.81 (-74.99, 158.13) |  |
| CT | -25.21 (-693.2, 644.84) | -200.38(-1090.12, 658.64) | CT | 3.37 (-82.05, 136.51) | -26.45 (-199.57, 124.74) | 9.1 (-97.21, 127.37) | -21.37(-138.68, 145.65) |  |
| HA | -5.17 (-480.64, 467.08) | -181.71(-511.62, 117.71) | 19.11 (-801.26, 835.81) | HA | -30.17 (-212.26, 82.68) | 5.9 (-104.07, 79.33) | -24.47(-115.05, 66.64) |  |
| PRP | 176.62(-689.71, 1069.63) | 0.98 (-670.6, 669.71) | 199.16(-885.22, 1319.74) | 182.47(-539.28, 933.98) | PRP | 35.72 (-103.27, 208.67) | 5.52 (-131.5, 213.38) |  |
| Saline | -43.92 (-713.52, 623.67) | -219.94(-1111.67, 644.21) | -18.53 (-967.21, 919.11) | -38.76(-857.48, 776.31) | -220.68(-1337.36, 872.53) | Saline | -30.47(-139.9, 116.33) |  |
| UCMSC | -4.63 (-828.06, 813.51) | -181.55(-937.83, 541.13) | 19.82(-1044.59, 1076.04) | 0.53 (-668.8, 669.4) | -182.52(-1194.18, 802.96) | 37.71 (-1021.27, 1098.07) | UCMSC |  |
| Six months and twelve months of WOMAC Stiffness | | | | | | | |  |
| ADMSC | ADMSC | -0.51 (-7.27, 4.29) | 21.76 (8.81, 33.67) | -0.02 (-4.3, 3.82) | -0.28 (-9.78, 7.19) | 0.36 (-3.29, 4.79) | 0.27 (-7.41, 7.53) |  |
| BMMSC | 0.12 (-7.96, 12.06) | BMMSC | 22.4 (11.14, 33.5) | 0.48 (-4.14, 6.55) | 0.2 (-6.01, 6.34) | 0.94 (-3.58, 7.87) | 0.75 (-6.63, 9.74) |  |
| CT | -22.19 (-37.36, -4.01) | -22.48 (-35.9, -8.77) | CT | -21.85 (-33.68, -9.19) | -22.19 (-34.65, -9.55) | -21.28 (-33.15, -8.24) | -21.59 (-34.58, -7.45) |  |
| HA | -0.1 (-7.2, 6.91) | -0.21 (-11.28, 7.02) | 22.12 (4.41, 36.75) | HA | -0.25 (-9.21, 7.08) | 0.39 (-4.22, 6.17) | 0.29 (-5.91, 6.54) |  |
| PRP | -0.11 (-12.83, 16.62) | -0.2 (-10.84, 10.55) | 22.27 (5.09, 39.12) | -0.02 (-12.16, 15.96) | PRP | 0.69 (-6.35, 10.45) | 0.54 (-8.91, 11.6) |  |
| Saline | -1.33 (-10.3, 7.88) | -1.53 (-12.65, 6.11) | 20.74 (2.85, 35.62) | -1.25 (-11.09, 8.86) | -1.27 (-17.47, 11.12) | Saline | -0.09 (-8.85, 7.44) |  |
| UCMSC | -0.39 (-13.17, 12.36) | -0.48 (-16.51, 11.61) | 21.87 (0.92, 39.28) | -0.3 (-11.03, 10.38) | -0.27 (-19.83, 15.32) | 0.95 (-13.71, 15.49) | UCMSC |  |
| Six months and twelve months of WOMAC Function | | | | | | | |  |
| ADMSC | ADMSC | -12.22 (-35.05, 18.86) | 6.94 (-29.09, 51.48) | 0.06 (-20.08, 19.27) | -10.89 (-46.94, 33.37) | -2.48 (-19.54, 18.91) | -2.88 (-39.64, 32.28) |  |
| BMMSC | 8.32 (-54.17, 74.99) | BMMSC | 19.19 (-10.55, 48.89) | 12.06 (-18.44, 33.93) | 1.46 (-28.14, 31.3) | 9.95 (-17.28, 32.58) | 9.31 (-35.27, 44.26) |  |
| CT | -10.89(-104.49, 86.72) | -19.22 (-89.84, 51.03) | CT | -6.99 (-51.13, 28.33) | -17.73 (-59.89, 23.88) | -9.33 (-50.46, 27.44) | -9.89 (-64.75, 35.32) |  |
| HA | -0.54 (-47.3, 44.66) | -8.65 (-70.87, 48.21) | 10.39 (-84.11, 99.59) | HA | -10.81 (-46.15, 33.48) | -2.42 (-25.09, 24.94) | -3.01 (-33.22, 26.71) |  |
| PRP | 7.06 (-87.31, 104.2) | -1.35 (-71.26, 69.34) | 17.84 (-81.13, 117.29) | 7.5 (-82.28, 101.99) | PRP | 8.55 (-32.63, 45.28) | 7.93 (-47.45, 52.64) |  |
| Saline | -1.84 (-60.55, 58.31) | -10.28 (-69.95, 48.21) | 8.98 (-83.93, 100.75) | -1.42 (-65.48, 65.67) | -8.95 (-101.76, 82.66) | Saline | -0.47(-41.85, 36.07) |  |
| UCMSC | 2.4 (-82.29, 85.51) | -5.84 (-99.98, 82.54) | 13.35 (-103.75, 125.56) | 3.01 (-67.34, 73.23) | -4.55 (-122.94, 108.03) | 4.29 (-92.34, 97.92) | UCMSC |  |
| Six months and twelve months of VAS Score | | | | | | | |  |
| \| ADMSC \| ADMSC \| -3.12(-17.23, 10.8) \| -17.01(-45.65, 13.67) \| 0.58(-22.36, 23.18) \| 1.88(-9.49, 15.44) \| 2.99(-16.89, 24.61) \| -2.92(-25.65, 19.99) \| 1.22(-8.56, 12.4) \| -14.02(-36.01, 9.81) \| \| --- \| --- \| --- \| --- \| --- \| --- \| --- \| --- \| --- \| --- \| \| BMMSC \| 11.43(-13.42, 37.5) \| BMMSC \| -13.87(-41.82, 16.19) \| 3.69(-14.12, 21.66) \| 5 (-4.33, 16.67) \| 6.11(-15.94, 29.83) \| 0.21(-17.61, 18.22) \| 4.34 (-9, 19.25) \| -10.92(-31.79,12.03) \| \| CSI \| 8.81(-37.17, 54.92) \| -2.6(-46.16, 39.77) \| CSI \| 17.59(-17.56, 50.36) \| 18.89(-7.96, 45.96) \| 20.07(-15.55, 54.98) \| 14.11(-21.09, 47.26) \| 18.24(-12.49, 48.36) \| 2.94(-16.14, 22.03) \| \| CT \| NA \| NA \| NA \| CT \| 1.27(-18.36, 23.27) \| 2.42(-25.82, 32.26) \| -3.49(-28.86, 21.79) \| 0.64(-21.4, 24.36) \| -14.6(-41.58, 14.71) \| \| HA \| -1.7(-23.28, 19.97) \| -13.1(-27.15, -0.26) \| -10.51(-51.45, 30.27) \| NA \| HA \| 1.16(-21.99, 23.54) \| -4.76(-26.68, 14.79) \| -0.68(-15.05, 13.15) \| -15.96(-35.2, 3.13) \| \| PLMSC \| NA \| NA \| NA \| NA \| NA \| PLMSC \| -5.92(-35.81, 22.34) \| -1.79(-19.62, 16.07) \| -17.09(-46.31, 13.17) \| \| PRP \| 10.4(-26.85, 48.62) \| -1.02(-28.89, 26.9) \| 1.59 (-48.95, 53.01) \| NA \| 12.09(-18.35, 43.5) \| NA \| PRP \| 4.13(-17.92, 27.73) \| -11.12(-38.45,18.21) \| \| Saline \| -11.28(-33.76,8.68) \| -22.75(-57.54, 8.92) \| -20.15(-72.06, 29.44) \| NA \| -9.61(-41.09, 19.57) \| NA \| -21.7(-66.91, 20.05) \| Saline \| -15.26(-38.8, 8.71) \| \| UCMSC \| 7.1(-28.92, 43.25) \| -4.33(-36.81, 27.08) \| -1.73 (-30.3, 27.01) \| NA \| 8.76(-19.87, 37.73) \| NA \| -3.31(-45.96, 38.66) \| 18.42(-22.22, 61.53) \| UCMSC \|   Six months and twelve months of WOMAC Pain | | | | | | | |  |
| ADMSC | ADMSC | -11.42 (-39.52, 11.77) | 12.1 (-29.75, 49.65) | -2.53 (-21.43, 16.58) | -11.28 (-52.95, 25.09) | 2.66 (-16.41, 21.63) | -4.63 (-40.08, 30.76) |  |
| BMMSC | 10.11 (-24.85, 52.94) | BMMSC | 23.55 (-6.81, 54.16) | 8.9 (-12.57, 35.04) | 0.09 (-29.58, 29.61) | 14.15 (-8.08, 40.93) | 6.73 (-29.15, 47.36) |  |
| CT | -13.32 (-67.35, 48.89) | -23.46 (-66.91, 20.03) | CT | -14.62 (-51.2, 26.2) | -23.45 (-66.32, 18.49) | -9.35 (-46.41, 31.88) | -16.77 (-63.52, 34.27) |  |
| HA | 1.98 (-26.51, 29.56) | -8.11 (-47.29, 22.43) | 15.3 (-44.72, 66.67) | HA | -8.74 (-49.52, 26.6) | 5.17 (-19.02, 29.36) | -2.12 (-31.68, 27.48) |  |
| PRP | 9.91 (-43.83, 72.09) | -0.08 (-43, 42.92) | 23.32 (-37.73, 84.71) | 7.91 (-42.38, 67.44) | PRP | 13.95 (-21.71, 55.12) | 6.64 (-39.23, 57.4) |  |
| Saline | -5.92 (-40.84, 31) | -16.06 (-55.35, 17.55) | 7.4 (-52.57, 60.79) | -7.92 (-45.82, 33.2) | -15.85 (-75.2, 37.12) | Saline | -7.28 (-45.59, 31.2) |  |
| UCMSC | 4.02 (-47.29, 54.8) | -5.98 (-65.25, 44.72) | 17.44 (-57.67, 83.05) | 2.07 (-40.68, 45.26) | -5.86 (-80.6, 59.03) | 9.99 (-49.61, 66.62) | UCMSC |  |

Note: The first half is the result of twelve months of follow-up and the second half is the result of six months of follow-up.

Table S2:SUCRA Results of Interventions for Each Outcome.

| Intervention | 6mo WOMAC  total | 12mo WOMAC total | 6mo WOMAC Stiffness | 12mo  WOMAC Stiffness | 6mo WOMAC Functional Limitation | 12mo WOMAC Functional Limitation | 6mo VAS | 12mo VAS | 6mo WOMAC pain | 12mo WOMAC pain | AE |
| --- | --- | --- | --- | --- | --- | --- | --- | --- | --- | --- | --- |
| BMMSC | 75.2 | 90.1 | 67.8 | 66.7 | 91.2 | 64.4 | 58.5 | 76.2 | 86.0 | 92.7 | 51.0 |
| ADMSC | 52.8 | 61.3 | 61.3 | 59.9 | 31.6 | 55.7 | 38.6 | 41.1 | 38.4 | 49.5 | 22.1 |
| UCMSC | 79.1 | 47.0 | 51.2 | 32.6 | 49.7 | 29.2 | 87.0 | 59.9 | 59.7 | 40.8 | 45.2 |
| PLMSC | NA | NA | NA | NA | NA | NA | 26.2 | NA | NA | NA | 4.0 |
| HA | 35.7 | 43.5 | 58.9 | 25.2 | 30.0 | 27.5 | 26.2 | 30.2 | 52.0 | 28.3 | 64.0 |
| Saline | 13.7 | 0.1 | 50.1 | NA | 50.6 | NA | 33.0 | 12.0 | 25.1 | 2.9 | 42.6 |
| PRP | 66.0 | 91.3 | 60.7 | 65.6 | 83.5 | 73.2 | 53.3 | 68.1 | 81.5 | 85.7 | 96.0 |
| CT | 27.4 | 16.7 | 0.0 | NA | 13.5 | NA | 36.3 | NA | 7.2 | NA | NA |
| CSI | NA | NA | NA | NA | NA | NA | 90.8 | 62.5 | NA | NA | 75.2 |

SUCRA surfaces under the cumulative ranking curve; WOMAC Western Ontario and McMaster Universities Osteoarthritis Index; VAS visual analogue scale; AE adverse event; BM-MSC, bone marrow mesenchymal stem cell; AD-MSC, adipose mesenchymal stem cell; UB-MSC, umbilical cord mesenchymal stem cell; HA, hyaluronic acid; PL-MSC, placenta-derived mesenchymal stem cells; PRP, platelet-rich plasma; CT, conservative treatment; CSI, Corticosteroid; mo month; NA no answer.

Figure.S1
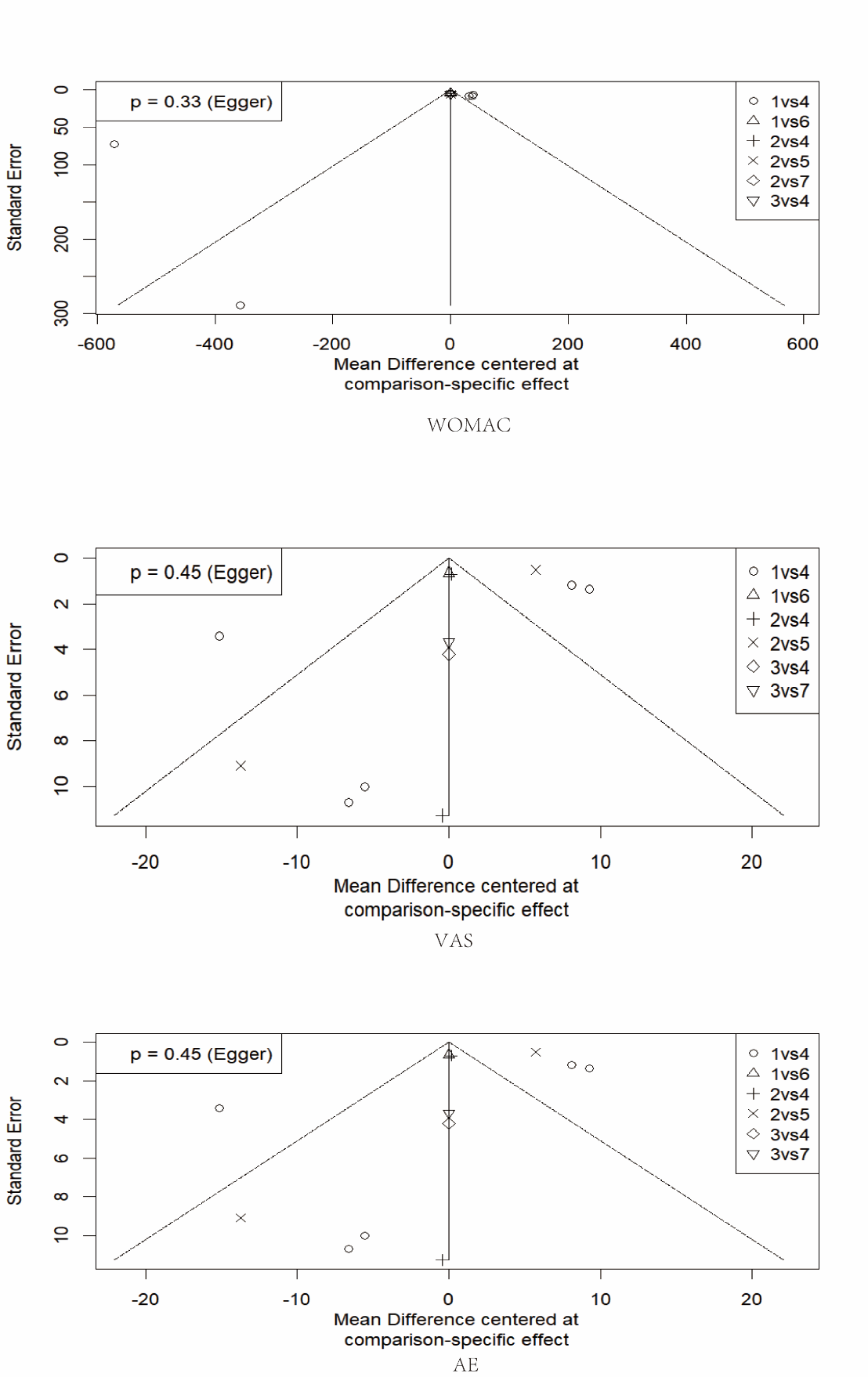
 Publication of bias evaluation results

WOMAC Western Ontario and McMaster Universities Osteoarthritis Index; VAS visual analogue scale; AE adverse event.
